# Entacapone alleviates muscle atrophy by modulating oxidative stress, proteolysis, and lipid aggregation in multiple mice models

**DOI:** 10.3389/fphys.2024.1483594

**Published:** 2024-12-09

**Authors:** Rong Zeng, Hanbing Xu, Mingzheng Wu, Xianlong Zhou, Pan Lei, Jiangtao Yu, Pinyi Wang, Haoli Ma, Yan Zhao

**Affiliations:** ^1^ Emergency Center, Hubei Clinical Research Center for Emergency and Resuscitaion, Zhongnan Hospital of Wuhan University, Wuhan, Hubei, China; ^2^ Department of Health Management, Renmin Hospital of Wuhan University, Wuhan, China; ^3^ Hubei Key Laboratory of Embryonic Stem Cell Research, Hubei Clinical Research Center for Umbilical Cord Blood Hematopoietic Stem Cells, Taihe Hospital, Hubei University of Medicine, Shiyan, Hubei, China; ^4^ Department of General Surgery, Wuhan Third Hospital, Tongren Hospital of Wuhan University, Wuhan, China; ^5^ Department of Biological Repositories, Zhongnan Hospital of Wuhan University, Wuhan, China

**Keywords:** entacapone, muscle atrophy, oxidative stress, proteolysis, lipid aggregation

## Abstract

**Background:**

Skeletal muscle atrophy significantly affects quality of life and has socio-economic and health implications. This study evaluates the effects of entacapone (ENT) on skeletal muscle atrophy linked with oxidative stress and proteolysis.

**Methods:**

C2C12 cells were treated with dexamethasone (Dex) to simulate muscle atrophy. Four murine models were employed: diaphragm atrophy from mechanical ventilation, Dex-induced atrophy, lipopolysaccharide (LPS)-induced atrophy, and hyperlipidemia-induced atrophy. Each model utilized entacapone (10 mg/kg), with sample sizes: Control (9), MV (11), MV + ENT (5) for diaphragm atrophy; Control (4), Dex (4), Dex + ENT (5) for Dex model; Control (4), LPS (4), LPS + ENT (5) for LPS model; and similar for hyperlipidemia. Measurements included muscle strength, myofiber cross-sectional area (CSA), proteolysis, oxidative stress markers [uperoxide dismutase 1 (SOD1), uperoxide dismutase 2 (SOD2), 4-hydroxynonenal (4-HNE)], and lipid levels.

**Results:**

Our findings confirm Dex-induced muscle atrophy, evidenced by increased expression of muscle atrophy-associated proteins, including Atrogin-1 and Murf-1, along with decreased diameter of C2C12 myotubes. Atrogin-1 levels rose by 660.6% (*p* < 0.05) in the Dex group compared to control, while entacapone reduced Atrogin-1 by 84.4% (*p* < 0.05). Similarly, Murf-1 levels increased by 365% (*p* < 0.05) in the Dex group and were decreased by 89.5% (*p* < 0.05) with entacapone. Dexamethasone exposure induces oxidative stress, evidenced by the upregulation of oxidative stress-related proteins Sod1, Sod2, and 4-HNE. Entacapone significantly reduced the levels of these oxidative stress markers, enhancing GSH-PX content by 385.6% (*p* < 0.05) compared to the Dex-treated group. Additionally, ENT effectively reduced the Dex-induced increase in MDA content by 63.98% (*p* < 0.05). Furthermore, entacapone effectively prevents the decline in diaphragm muscle strength and myofiber CSA in mice. It also mitigates diaphragm oxidative stress and protein hydrolysis. Additionally, entacapone exhibits the ability to attenuate lipid accumulation in the gastrocnemius muscle of hyperlipidemic mice and alleviate the reduction in muscle fiber CSA.

**Conclusion:**

Our findings suggest that entacapone is a promising therapeutic candidate for muscle atrophy, functioning through the reduction of oxidative stress, proteolysis, and lipid aggregation. Future research should explore the underlying mechanisms and potential clinical applications of entacapone in muscle-wasting conditions.

## Introduction

Muscle atrophy significantly impacts body movement, thermogenesis, metabolism, and overall health maintenance. It can be caused by various factors, including denervation, disuse, fasting, disease, and aging (Li et al., 2017; Liu et al., 2021; Wang et al., 2023). The consequences of muscle atrophy extend beyond impairing daily functioning; they also influence prognosis and mortality rates (Wolfe, 2006). Key indicators of muscle atrophy include Atrogin-1 (also known as MAFBX) and muscle-specific RING-finger 1 (Murf-1) (Bodine et al., 2001).

The pathogenesis of muscle atrophy involves several molecular mechanisms, with oxidative stress and inflammation playing critical roles. Reactive oxygen species (ROS) generated from oxidative stress activate proteolytic pathways and inhibit protein synthesis (Powers et al., 2020). Inflammation and oxidative stress reinforce each other, creating a vicious cycle where inflammation exacerbates oxidative stress, which in turn promotes further inflammation. Such mechanisms are crucial for understanding muscle atrophy and have prompted research into antioxidant and anti-inflammatory therapies. While some therapies have shown potential in alleviating muscle atrophy, challenges remain in translating these findings into clinical practice (Dridi et al., 2020; Huang et al., 2023; Li et al., 2022a; Huang et al., 2022).

External factors, such as dexamethasone, Lipopolysaccharide (LPS), and hyperlipidemia, have also been demonstrated to induce muscle atrophy (Altun et al., 2010; Shen et al., 2019; Doyle et al., 2011; Picard et al., 2012). Prolonged mechanical ventilation (MV) can lead to ventilator-induced diaphragmatic dysfunction (VIDD), characterized by significant diaphragmatic muscle fiber atrophy and weakness, complicating patient recovery (Goligher et al., 2018; Sklar et al., 2020). Factors contributing to VIDD include not only mechanical stress but also systemic inflammation and oxidative stress.

A pivotal player in muscle atrophy is the transcription factor Foxo1 (Kim et al., 2024; Yang et al., 2024). Elevated Foxo1 activity is linked to various stressors and promotes the expression of genes associated with muscle degradation (Zufiría et al., 2024; Liu et al., 2020). Consequently, inhibiting Foxo1 activity emerges as an effective strategy for preventing muscle atrophy in various pathological conditions (Smuder et al., 2015). Entacapone (ENT), an FDA-approved drug commonly used for managing Parkinson’s disease, has been shown to inhibit Foxo1 activity (Peng et al., 2019), and may influence muscle metabolism through additional pathways (Li et al., 2015). This makes entacapone a compelling candidate for mitigating skeletal muscle atrophy.

This study aims to explore the effects of entacapone as a potential therapeutic strategy against muscle atrophy. By investigating the mechanisms of muscle atrophy induced by diverse stimuli, we aim to enhance our understanding of its biological underpinnings and provide a foundation for novel therapeutic approaches. We employ an *in vitro* model alongside four *in vivo* models to comprehensively evaluate the effects of entacapone on skeletal muscle atrophy. The *in vitro* model allows for an initial assessment of entacapone’s direct effects on skeletal muscle cells, establishing a basis for subsequent *in vivo* investigations.

The hospitalization or mechanical ventilation-induced muscle atrophy model simulates the real-life experiences of critically ill patients suffering from VIDD, enabling us to evaluate entacapone’s protective effects in this specific context. The drug-induced muscle atrophy model examines whether entacapone can mitigate muscle damage caused by other medications, providing crucial insights into its safety and potential side effects. Additionally, the inflammation-induced muscle atrophy model reveals entacapone’s protective mechanisms against muscle damage mediated by inflammatory processes. Lastly, the muscle atrophy model associated with hyperlipidemia offers insights into how entacapone affects muscle wasting in the context of metabolic dysregulation.

Through the integration of these models, we aim to deliver a comprehensive assessment of entacapone’s effects on muscle atrophy across diverse pathological conditions, thereby laying a robust foundation for its clinical application.

## Methods and materials

### Cell culture and differentiation

C2C12 cells were obtained from ATCC and maintained in Dulbecco’s modified Eagle’s medium (DMEM) supplemented with 15% fetal bovine serum, 1% penicillin-streptomycin, and 0.1% tetracycline. The cells were cultured at 37°C with 5% CO_2_ until reached a density of approximately 70%–80%. Subsequently, C2C12 cell differentiation was induced by treating the cells with 2% horse serum and 1% penicillin-streptomycin. The horse serum was refreshed daily. On the fourth day, the cells were treated with 100 μM entacapone (MCE, dissolved in DMSO) or the corresponding vehicle control. On the sixth day, C2C12 myotubes were cultured with DMEM with 2% horse serum and 50 μM dexamethasone (Sigma, D4902) for 24 h. Each group underwent four distinct experiments *in vitro* to ensure reliability and reproducibility of the results.

### Animals

Male C57BL/6J mice and APOE^−/−^ mice (C57BL/6J background), aged 6–8 weeks, were purchased from GemPharmatech Co., Ltd. The mice were housed under controlled conditions (22°C–25°C, 12-h light/dark cycle, 45%–55% humidity) with *ad libitum* access to food and water. All animal experiments adhered to the National Institutes of Health guide for the care and use of laboratory animals and were approved by the Bio-Safety Level III Animal Laboratory of Wuhan University (Wuhan, China).

#### Study 1: Investigating entacapone’s role in the diaphragm during mechanical ventilation

Animals were randomly assigned into three groups: (1) spontaneous breath group (SB); (2) mechanical ventilation group (MV): C57BL/6 J mice were mechanically ventilated for 12 h under continuous anesthesia (Matecki et al., 2016); and (3) mechanical ventilation and entacapone treatment group (MV + Ent): mice were mechanically ventilated for 12 h following an intraperitoneal injection of entacapone (HY-14280, MCE, 10 mg/kg, dissolved in 40% PEG300, 10% DMSO, 5% Tween-80, in 45% saline) (Luo et al., 2021). The 12-h mechanical ventilation duration was selected based on previous studies indicating that this time frame effectively models diaphragm muscle atrophy. The initial numbers were: Control (n = 11), MV (n = 11), MV + Ent (n = 5). However, during the experiment, two mice in the Control group were excluded due to complications arising from anesthesia, resulting in final group sizes of Control (n = 9), MV (n = 11), MV + Ent (n = 5). The unbalanced distribution of sample sizes resulted from the exclusion of animals due to health complications and adverse reactions during the experiments, which were unrelated to the experimental treatments. This variability may influence statistical analyses, and we acknowledge this as a limitation of the study.

#### Study 2: Investigating entacapone in a dexamethasone-induced muscle atrophy model

The animals were randomly divided into three groups: (1) Control group (Ctrl): mice were intraperitoneally injected with solvent daily for 10 days; (2) dexamethasone group (Dex): mice were intraperitoneally injected with dexamethasone (25 mg/kg, D4902, Sigma, dissolved in sterile saline) daily for 12 days (Wang et al., 2021); and (3) dexamethasone and entacapone group (Dex + Ent): mice were intraperitoneally injected with entacapone (10 mg/kg) and dexamethasone (25 mg/kg) daily for 10 days. The initial numbers were: Control (n = 5), Dex (n = 5), Dex + Ent (n = 5). During the study, 1 mouse from the Control group and one from the Dex group were removed due to poor health unrelated to the experimental, leading to final counts of Control (n = 4), Dex (n = 4), Dex + Ent (n = 5).

#### Study 3: Evaluating entacapone in an LPS-Induced muscle atrophy model

The animals were randomly divided into three groups: (1) Control group (Ctrl, n = 4): mice were intraperitoneally injected with saline; (2) LPS group (LPS): mice were intraperitoneally injected with lipopolysaccharides (LPS) (10 mg/kg, Sigma, dissolved in sterile saline) (Li et al., 2024); (3) LPS and entacapone group (LPS + Ent): mice were intraperitoneally injected with entacapone (10 mg/kg) and LPS (10 mg/kg). The mice were sacrificed 72 h after the start of treatment. The initial groups comprised Control (n = 4), LPS (n = 5), LPS + Ent (n = 5). However, 1 mouse from the LPS group were excluded due to severe adverse reactions to LPS, resulting in final numbers of Control (n = 4), LPS (n = 4), and LPS + Ent (n = 5).

#### Study 4: Assessing the effects of APOE deficiency and entacapone on muscle mass and function

Wild-type or APOE-deficient C57BL/6 mice were divided into three groups: (1) wild-type C57BL/6 mice group injected with solvent (WT): wild-type C57BL/6 mice were intraperitoneally injected with solvent; (2) APOE group (APOE): mice were intraperitoneally injected with normal saline; (3) APOE and entacapone group (APOE + Ent): APOE-deficient C57BL/6 mice were intraperitoneally injected with entacapone (10 mg/kg). Initially, the groups comprised WT (n = 4), APOE (n = 5), and APOE + Ent (n = 5). One mouse from the APOE group was removed due to pre-existing health conditions not related to the study intervention, resulting in final group sizes of WT (n = 4), APOE (n = 4), and APOE + Ent (n = 5). The APOE-deficient model was selected due to its significance in studying lipid metabolism and its role in muscle atrophy, as it serves as a model for hyperlipidemia, providing insights into the underlying mechanisms of these processes (Vaziri et al., 2010; Ghiselli et al., 1981).

At the conclusion of the experiments, the diaphragm and gastrocnemius muscle samples were collected for biochemical analysis, muscle strength measurements.

### Collection of samples for analysis

At the conclusion of the experiments, specific tissues and blood samples were collected for various analyses: Muscle Tissue Collection: The diaphragm and gastrocnemius muscle samples were excised immediately following sacrifice and rinsed in cold PBS. For biochemical analysis, muscle tissues were snap-frozen in liquid nitrogen and stored at −80°C. For histological analysis, muscle samples were fixed in 4% paraformaldehyde and then processed for paraffin embedding.

#### Blood collection

Blood samples were collected via cardiac puncture. The blood was allowed to clot at room temperature for 30 min, then centrifuged at 3,000 rpm for 10 min to separate the serum. The serum was carefully collected and stored at −80°C for lipid analysis.

#### Histological processing

Lung tissues were collected, fixed in 4% paraformaldehyde, dehydrated, and embedded in paraffin. Sections were cut for H&E staining and Oil-Red O staining to evaluate histological changes.

### Mechanical ventilation

After weighing, the mice were intraperitoneally anesthetized with 1% pentobarbital sodium. The shaved area was disinfected, and the mice were then connected to a small animal ventilator (VentElite, Harvard Apparatus, United States). The tidal volume was set at 10 mL/kg, with a positive end-expiratory pressure (PEEP) of 2 cm H_2_O. The respiratory rate (RR) was maintained at 150 breaths per minute (Li et al., 2022b; Mrozek et al., 2012). To maintain their body temperature at 37°C, the mice were placed on a temperature-controlled blanket. Anesthesia was continuously administered to the mice using a micropump throughout the duration of the experiment.

### Muscle force measurements

After collection, the diaphragm was promptly immersed in a Krebs-Henseleit solution (pH 7.4, 25°C) that had been equilibrated with a gas mixture of 95% O_2_ and 5% CO_2_. The diaphragm was allowed to equilibrate in this solution for 15 min to ensure uniform adaptation to the environment. Subsequently, the muscles were secured onto the equipment and connected to an experimental instrument system (Aurora Scientific Inc, 1200A, Canada) for the measurement of diaphragm muscle strength (Qaisar et al., 2019; Ham et al., 2022). Muscle contractility was assessed by delivering electrical stimulations at varying frequencies (10 Hz, 20 Hz, 40 Hz, 60 Hz, 80 Hz, 100 Hz, 120 Hz, and 140 Hz) using a stimulation voltage of 15V, a pulse duration of 2 milliseconds, and a stimulation interval of 2 min. Each stimulation lasted consistently across frequencies, with a 1-min rest interval between stimuli to allow for recovery. The isometric contraction force was recorded in Newtons (N) during each stimulation. To calculate the muscle’s CSA, the diaphragm’s mass was measured, and CSA was derived using the formula: muscle mass divided by the product of its optimal initial length (Lo) and an assumed muscle density of 1.056 g/cm³. The specific force (in Newtons per square centimeter, N/cm^2^) was calculated by dividing the recorded muscle force by the CSA (in cm^2^). This comprehensive methodology provided an accurate assessment of the diaphragm’s force output under varying stimulation conditions, yielding critical data for further physiological research.

### Measurement of serum lipid

The levels of total cholesterol (TC), high-density lipoprotein cholesterol (HDL-C), triglycerides, and low-density lipoprotein cholesterol (LDL-C) in the mice were quantified using an automated chemistry analyzer (Cobas, MiraPlus, Roche Diagnostics) according to the manufacturer’s instructions.

### Histological analysis

The lung tissues underwent fixation, dehydration, and subsequent paraffin embedding. Afterward, the tissues were sectioned and subjected to routine Hematoxylin and Eosin (H&E) staining. H&E-stained sections were used primarily for qualitative assessment of histopathological changes. The focus of this analysis was to visually identify structural alterations rather than to quantify specific parameters.

### Oil-Red O staining

To evaluate the influence of lipids on muscle, frozen sections were fixed with 4% paraformaldehyde for 10 min. Following fixation, the sections were washed with 60% isopropanol and stained with Oil Red O at 37°C for 15 min to visualize lipid droplets within the cytoplasm. Subsequently, nuclear counterstaining was carried out using hematoxylin. Lipid Accumulation Measurement: Lipid accumulation was assessed through visual observation of Oil Red O-stained areas.

### CCK-8 assay

C2C12 cells were seeded at a density of 2 × 10^3^ cells per well in 96-well plates. After inducing differentiation for 4 days to form myotubes, the cells were treated with different concentrations of entacapone (50, 100, 150, 200, 250 μM) for 48 h. Additionally, varying concentrations of dexamethasone (50, 100, 150, 200, 250 μM) were added during the last 24 h of treatment. Following the manufacturer’s instructions, the CCK-8 reagent was mixed with the cell culture medium. After a 2-h incubation, the absorbance at 450 nm was measured using a microplate reader. Cell viability was calculated according to the provided instructions.

### Immunofluorescence

C2C12 cells and mouse muscle sections were fixed in 4% paraformaldehyde for 25 min. Subsequently, permeabilization was performed using 0.5% Triton X-100 for 15 min, followed by blocking with 5% BSA for 1 h. For C2C12 cells, the primary antibody used was myosin heavy chain (Abclonal; A4963, 1:200). For mouse muscle sections, specific primary antibodies included anti-slow-twitch myosin heavy chain (NOQ7.5.4D, Abcam; ab11083, 1:200), anti-fast-twitch myosin heavy chain (MY-32, Abcam; ab51263, 1:200), and anti-laminin (Abcam; ab11575, 1:500). The samples were incubated overnight at 4°C with the respective antibodies. Afterward, the samples were incubated with the appropriate secondary antibody for 1 h at room temperature. Following PBS washing steps, the nuclei were stained with DAPI. Finally, the samples were sealed, and images were acquired using a fluorescence microscope (Olympus, IX73, Japan).

### Western blotting

Protein samples were extracted by lysing muscle tissue or cells using RIPA buffer (Solarbio, China) supplemented with protease and phosphatase inhibitors. For each sample type (cells, diaphragm, gastrocnemius), 30 µg of total protein was loaded into the gel. Following determination of protein sample concentrations, equal amounts of protein samples were subjected to SDS-polyacrylamide gel electrophoresis and subsequently transferred to an NC membrane at 300 mA for 1 h. The membranes were then blocked with 5% nonfat milk. Specific primary antibodies, including Atrogin-1 (Abcam; ab168372, 1:1,000), Murf-1 (Abcam; ab172479, 1:1,000), 4HNE (Abcam; ab46545, 1:1,000), GAPDH (Abcam; ab181602, 1:1,000), superoxide dismutase 1 (SOD1) (Abclonal; A0274, 1:2000), and superoxide dismutase 2 (SOD2) (Abclonal; A19576, 1:2000) were incubated with the membranes overnight at 4°C. After washing with TBST, the membranes were incubated with corresponding secondary antibodies (goat anti-rabbit IgG, Abcam; ab6721, 1:2000) for 1 h at room temperature, followed by further washing with TBST. Chemiluminescent detection was performed using the ECL reagent (Thermo Scientific; 32,106) according to the manufacturer’s instructions. Blot quantification was carried out using Image Lab Software (Bio-Rad), normalizing band intensity to GAPDH as a loading control.

### Quantitative real-time polymerase chain reactions (qPCR)

Total RNA was extracted from cells and muscle tissue using Trizol Reagent following the manufacturer’s instructions. The concentration and purity of RNA were determined using a NanoDrop spectrometer (Thermo Fisher Scientific, Inc.). Total RNA was prepared at a concentration of 1 μg/μL for cDNA synthesis using the ABScript II cDNA First-Strand Synthesis Kit (RK20400). Quantitative Real-time PCR was conducted on a QuantStudio 1 Plus system (Applied Biosystems) using the Universal SYBR Green Fast qPCR Mix (2X) (Abclonal, RK21203) and analyzed with QuantStudio TM Design and Analysis Software v1.0.0 (United States). The relative gene expression levels were calculated by the 2^−ΔΔCT^ method using Glyceraldehyde 3-phosphate dehydrogenase (GAPDH) as the internal control. The primer sequences used in the study are reported below:

Atrogin-1 (F-TTCAGCAGCCTGAACTACGA; R-AGTATCCATGGCGCTCCTTC), Murf-1 (F-GCGTGACCACAGAGGGTAAA; R-CTCTGCGTCCAGAGCGTG) and GAPDH (F-AGGTCGGTGTGAACGGATTTG; R-TGTAGACCATGTAGTTGAGGTCA).

### Measurements of malondialdehyde (MDA) levels and glutathione peroxidase (GSH-PX) activities

The levels of MDA and GSH-PX activity in cells were determined using assay kits (MDA assay kit, Nanjing Jiancheng Bioengineering Institute; Cat. No. A003-1-1; GSH-PX assay kit, Nanjing Jiancheng Bioengineering Institute; Cat. No. A005-1-1) following the manufacturer’s instructions.

### Statistical analysis

Data were presented as mean ± standard error of the mean (SEM). The normality of the data was assessed using the Shapiro-Wilk test. We performed statistical analyses using one-way analysis of variance (ANOVA), acknowledging that the unbalanced sample sizes could affect the type I and type II error rates. We recommend cautious interpretation of the results, particularly where group sizes differ significantly. Statistical comparisons between groups were conducted using ANOVA. When significant differences were found, *post hoc* analysis was performed using Tukey’s test for multiple comparisons between groups. For comparisons between two groups, unpaired two-tailed Student’s t-test was employed. All statistical analyses were performed using Prism 8.0.1 software (GraphPad Software, Inc., CA, United States). A two-tailed *p*-value below 0.05 was deemed statistically significant.

## Results

### Entacapone mitigated survival rate of dex-induced myotube atrophy *in vitro*

**FIGURE 1:**
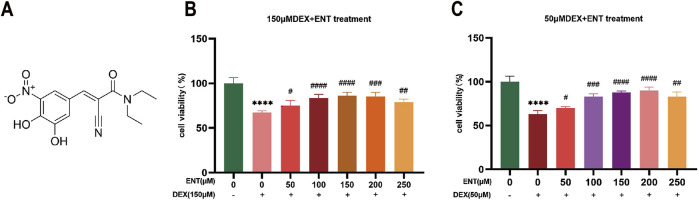
Entacapone improves Dex-induced muscle atrophy in C2C12 myotubes. **(A)** Chemical structure of ENT. **(B)** Cell viability of C2C12 myotubes treated with various concentrations of ENT (50, 100, 150, 200, 250 μM) for 48 h, along with 50 μM Dex during the final 24 h. **(C)** Viability of C2C12 myotubes treated with different concentrations of ENT (50, 100, 150, 200, 250 μM) for 48 h, along with 150 μM Dex during the last 24 h. Statistical analyses were performed using one-way ANOVA with n = 4 per group. Date was expressed as the mean ± SEM. *p < 0.05, **p < 0.01, ***p < 0.001, ****p < 0.0001 indicate significant differences between the Control and Dex groups. ^#^ *p* < 0.05, ^##^ *p* < 0.01, ^###^ *p* < 0.001, ^####^ *p* < 0.0001 indicate significant differences between the ENT and Dex groups. Dex, dexamethasone; Murf-1, muscle ring finger 1; ENT, entacapone.

To investigate the impact of entacapone on cellular myotube atrophy *in vitro*, various concentrations of ENT (50, 100, 150, 200, and 250 μM) were added to myotubes to prevent dexamethasone (Dex)-induced cell death at concentrations of 50 and 150 μM (Figures 1A, B).

### Entacapone reduced dexamethasone-induced myotube atrophy

**FIGURE 2:**
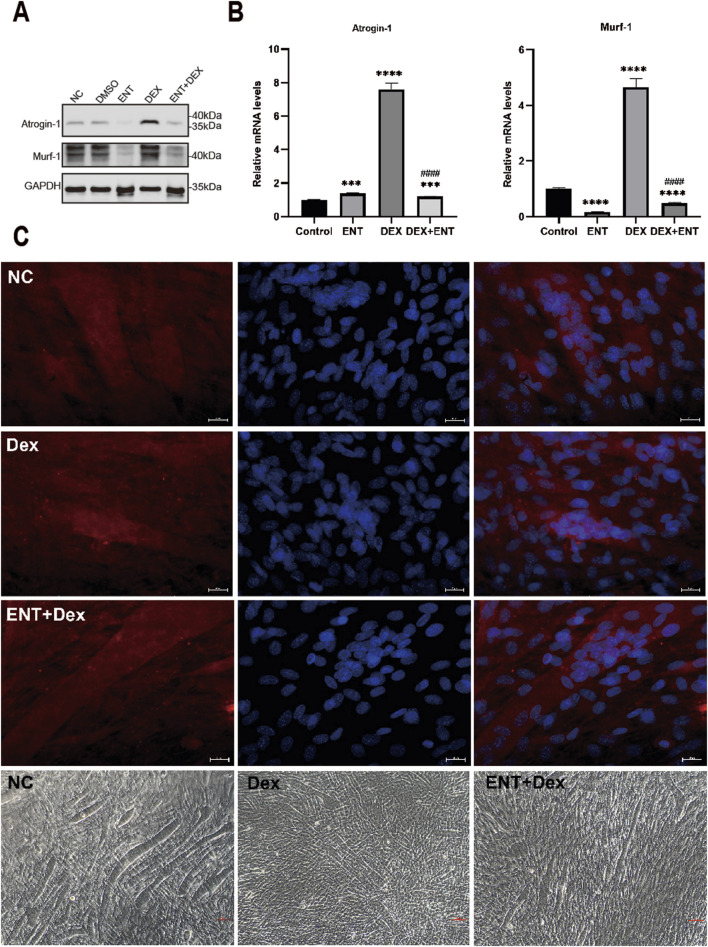
Representative pictures of C2C12 myotubes treated with DMSO or 50 µM Dex, 100 µM ENT. **(A)** Protein levels of Atrogin-1 and Murf-1 in C2C12 myotubes treated with 50 μM Dex and 100 μM ENT, with GAPDH serving as a loading control. **(B)** qRT-PCR analysis of Atrogin-1 and Murf-1 in C2C12 myotubes treated with 50 μM Dex and 100 μM ENT. **(C)** Immunofluorescence staining and phase-contrast microscope images of C2C12 myotubes revealed that Dex induced muscle atrophy, as evidenced by a reduction in myotube diameter, while ENT mitigated Dex-induced muscle atrophy in C2C12 myotubes. Each experiment was conducted with a sample size of n = 4 per group. NC represents the control group, Dex refers to dexamethasone, ENT + Dex indicates entacapone + dexamethasone. The scale bar represents 25 μm.

Furthermore, the addition of 100 μM ENT was found to effectively protect against the upregulation of Atrogin-1 and Murf-1 protein levels induced by 50 μM Dex (Figure 1C). Specifically, Atrogin-1 levels significantly increased by 660.6% (*p* < 0.05) in the Dex-treated group compared to the control, while entacapone treatment reduced Atrogin-1 levels by 84.4% (*p* < 0.05) compared to the Dex group. Similarly, Murf-1 levels rose by 365% (*p* < 0.05) in the Dex group compared to control and were decreased by 89.5% (*p* < 0.05) with entacapone treatment. Similar observations were made regarding the attenuation by 100 μM ENT of the upregulation of Atrogin-1 and Murf-1 mRNA levels induced by 50 μM dexamethasone (Figures 2A, B). Collectively, these findings indicate that ENT demonstrates potential in ameliorating Dex-induced muscle atrophy in C2C12 myotubes. To investigate the potential protective effect of ENT against myotube atrophy *in vitro*, we examined the impact of ENT on C2C12 myotube diameter, as shown in Figure 2C. Dex treatment significantly reduced the diameter of C2C12 myotubes, which was subsequently restored by the addition of ENT. The microscopic images were consistent with the immunofluorescence results. Notably, ENT effectively protected against Dex-induced myotube atrophy.

### Entacapone mitigated dex-induced atrophy in C2C12 myotubes

**FIGURE 3:**
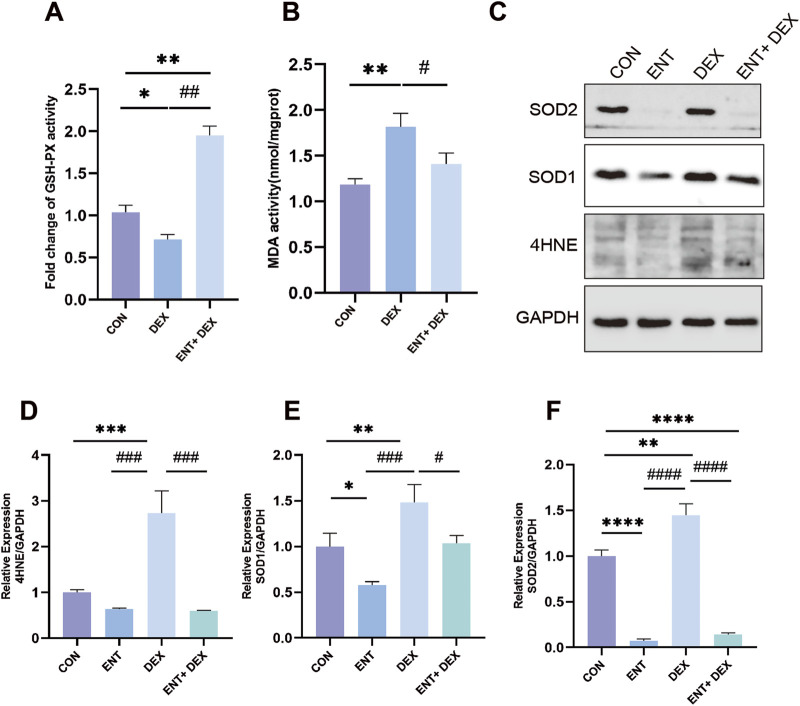
ENT confers protection against Dex-induced muscle atrophy by mitigating oxidative stress in C2C12 myoblasts. The effects of ENT on the activity of GSH-PX **(A)** and MDA content **(B)** were evaluated in C2C12 myoblasts treated with 50 µM Dex, with or without ENT for 48 h. **(C–F)** ENT attenuated the Dex-induced increases in the expression of Murf-1, Atrogin-1, 4-HNE, SOD1, and SOD2 in C2C12 myotubes. Date was expressed as the mean ± SEM. *p < 0.05, **p < 0.01 versus NC group; ^#^ *p* < 0.05, ^##^ *p* < 0.01 versus Dex group.

Dex treatment resulted in a significant decrease in GSH-PX content and an increase in MDA content, indicating elevated oxidative stress in C2C12 myotubes (Figures 3A, B). Specifically, treatment with 50 μM Dex led to a marked increase in MDA levels, a well-known marker of lipid peroxidation, which suggests enhanced oxidative damage to cell membranes. However, ENT effectively reduced this Dex-induced increase in MDA content by 63.98% (*p* < 0.05), demonstrating its potential protective role against lipid oxidative stress. Additionally, ENT significantly increased GSH-PX content compared to the Dex-treated group by 385.6% (*p* < 0.05). GSH-PX is a key antioxidant enzyme that plays a critical role in reducing oxidative damage by catalyzing the reduction of hydrogen peroxide and organic peroxides. This increase suggests that ENT not only mitigates oxidative damage but also enhances the antioxidant defense system in C2C12 myotubes. To assess the efficacy of ENT in countering Dex-induced oxidative stress, we further analyzed additional oxidative stress-related markers, including 4-HNE, SOD1, and SOD2. Dex treatment significantly increased the levels of 4-HNE-modified proteins, indicating heightened lipid peroxidation. ENT treatment effectively attenuated this increase, reinforcing its role in reducing oxidative damage. Additionally, we observed that ENT inhibited the Dex-induced upregulation of SOD1 and SOD2, which are important antioxidant enzymes that help mitigate ROS levels (Figures 3C–F). The downregulation of these enzymes suggests that while Dex induces oxidative stress, ENT may help restore the balance by modulating these protective pathways. Collectively, these findings indicate that Dex exposure significantly upregulates markers of oxidative stress, while ENT exerts a protective effect against Dex-induced oxidative stress and muscle atrophy in C2C12 myotubes.

### Entacapone protected mice against ventilation-induced diaphragm dysfunction and dexamethasone-induced muscle dysfunction

**FIGURE 4:**
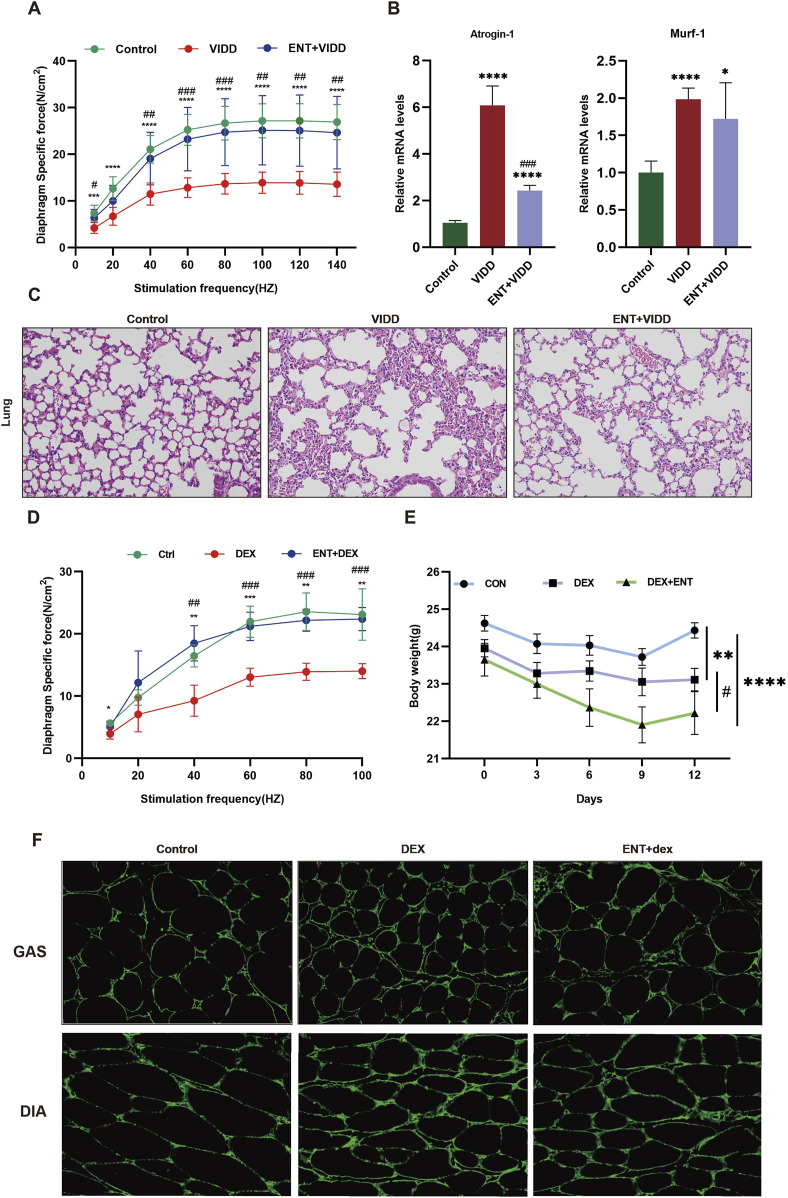
ENT protects mice against ventilation-induced diaphragm dysfunction and Dex-induced muscle atrophy. **(A)** Diaphragm force-frequency relationship in VIDD. **(B)** Protein expression levels of Atrogin-1 and Murf-1. **(C)** H&E staining of the lungs. **(D)** Diaphragm force-frequency relationship in Dex-induced muscle atrophy. **(E)** Changes in mouse body weight. **(F)** ENT protects against Dex-induced diaphragm and gastrocnemius muscle atrophy. VIDD, ventilator-induced diaphragm dysfunction group; ENT, entacapone; Dex, dexamethasone group. *p < 0.05, **p < 0.01, ***p < 0.001, ****p < 0.0001 vs. Ctrl; ^#^ *p* < 0.05, ^##^ *p* < 0.01, ^###^ *p* < 0.001 vs. Dex.

After 12 h of continuous MV, diaphragm muscle strength exhibited a significant decrease compared to the control group. Treatment with ENT substantially mitigated this decrease (Figure 4A). Additionally, MV resulted in elevated expression levels of Atrogin-1 and Murf-1; however, ENT treatment effectively reduced the ventilation-induced upregulation of these markers (Figure 4B). H&E staining of lung tissue revealed alveolar septal thickening, alveolar hemorrhage, and alveolar collapse in the mechanically ventilated group. Notably, these pathological changes were significantly alleviated following the administration of ENT (Figure 4C). High doses of Dex led to a decline in diaphragm strength, but diaphragm contractility in mice was enhanced in the ENT + Dex group compared to the Dex group (Figure 4D). Mice subjected to Dex-induced muscle atrophy experienced substantial weight loss when compared to the control group. Mice in the ENT + Dex group also exhibited weight loss relative to both the control group and the Dex group alone (Figure 4E). The CSA of both the gastrocnemius and diaphragm displayed a reduction in the Dex group when compared with the control group. However, ENT effectively safeguarded against this reduction (Figure 4F).

### Entacapone modulates molecular mechanisms involved in LPS-Induced muscle atrophy

**FIGURE 5:**
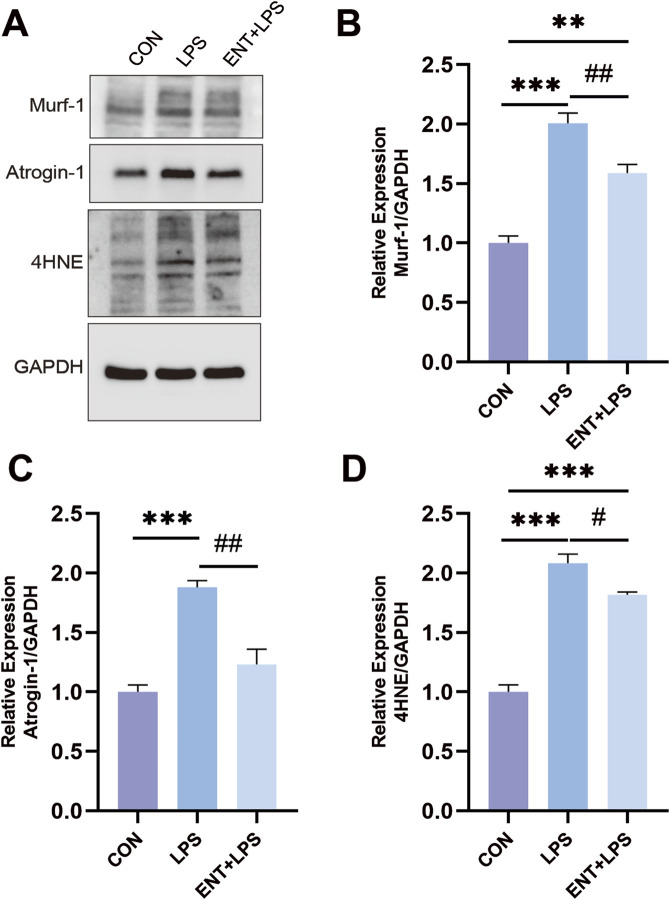
Effects of ENT on LPS-induced muscle atrophy. **(A–D)** The diaphragm’s Murf-1, Atrogin-1, and 4-HNE expression levels were analyzed using Western blotting. The findings demonstrated that the LPS group exhibited an elevation in diaphragmatic oxidative stress and proteolysis levels compared to the Control group. However, ENT treatment was able to mitigate this upregulation of oxidative stress and proteolytic activity. **p < 0.01, ***p < 0.001 vs. control; ^#^ *p* < 0.05, ^##^ *p* < 0.01 vs. LPS.

To investigate the effects of LPS-induced muscle atrophy and the protective role of ENT against inflammation-related muscle atrophy, we examined the expression levels of muscle atrophy and oxidative stress markers: Atrogin-1, Murf-1, and 4-HNE. As anticipated, LPS-induced muscle atrophy led to a notable elevation in the levels of Atrogin-1, Murf-1, and 4-HNE (*p* < 0.05), indicating the activation of pathways associated with muscle degradation and oxidative stress. However, in the ENTLPS group, these levels exhibited a significant reduction compared to the LPS group (*p* < 0.05) (Figure 5). This reduction suggests that ENT effectively mitigates oxidative stress, thereby exerting a protective influence on the molecular mechanisms involved in muscle atrophy.

### Entacapone reduced muscle atrophy associated with hyperlipidemia

**FIGURE 6:**
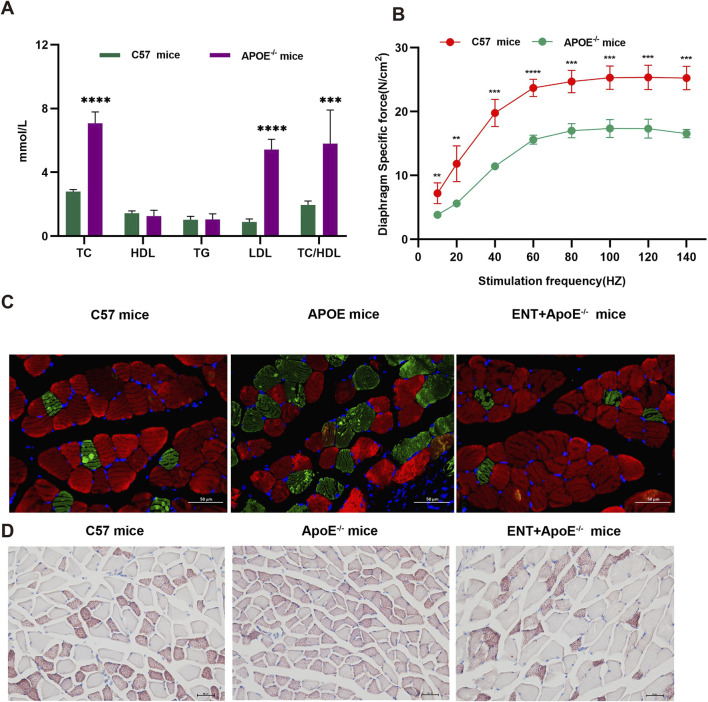
Effect of ENT on skeletal muscle in APOE^−/−^ hyperlipidemic mice. **(A)** Blood lipid concentrations in C57 and APOE^−/−^ mice. **(B)** Diaphragm force-frequency relationship in C57 and APOE^−/−^ mice. **(C)** Representative immunofluorescence staining images of slow-twitch and fast-twitch fibers in the gastrocnemius. **(D)** Oil-red O staining of the gastrocnemius was used to evaluate lipid accumulation in C57 and APOE^−/−^ mice. n = 6–10 per group. TC, total cholesterol; HDL-C, high-density lipoprotein cholesterol; TG, triglycerides; LDL-C, low-density lipoprotein cholesterol. Scale bar, 50 μm.

To evaluate the impact of hyperlipidemia on muscle atrophy and the potential effects of ENT on the muscles of mice with hyperlipidemia, we employed APOE^−/−^ mice and measured blood lipid levels. The results indicated that APOE^−/−^ mice exhibited higher levels of TC, LDL, and TC/HDL compared to C57 mice (Figure 6A). Furthermore, muscle strength was reduced in hyperlipidemic mice relative to C57 mice (Figure 6B). Concurrently, immunofluorescence analysis of the gastrocnemius muscle revealed a smaller CSA of gastrocnemius myofibers in APOE^−/−^ mice compared to C57 mice, suggesting myofiber atrophy. Notably, the administration of ENT mitigated this myofiber atrophy (Figure 6C). Lipid content was assessed through Oil Red O staining, which corroborated the reduction in gastrocnemius muscle CSA in APOE^−/−^ mice and demonstrated that ENT treatment protected against muscle fiber atrophy. Additionally, APOE^−/−^ mice exhibited more lipid droplet accumulation in muscle compared to C57 mice, while treatment with ENT significantly alleviated lipid aggregation in the gastrocnemius muscle of APOE^−/−^ mice (Figure 6D). Taken together, our findings demonstrate that ENT treatment confers a protective effect against muscle atrophy associated with hyperlipidemia.

## Discussion

Despite the significant socioeconomic and public health burden imposed by muscle atrophy (Ye et al., 2022; Cruz-Jentoft et al., 2019), treatments and drugs for various forms of muscle wasting are still in the developmental stages. Consequently, there is an urgent need to identify effective interventions and therapeutics capable of preventing or ameliorating muscle atrophy. Prior research has highlighted that the management of muscle wasting primarily involves approaches targeting antioxidant activity, anti-inflammatory mechanisms, protein synthesis promotion, and proteolysis inhibition (Huang et al., 2022).

Entacapone commonly used in clinical settings for the treatment of Parkinson’s disease, has been found to counteract oxidative stress (Helkamaa et al., 2003) and inflammation (Luo et al., 2021). This study explores the efficacy of ENT in addressing muscle atrophy, offering innovative strategies for prevention and treatment across various models of muscle wasting.

The pathogenesis of muscle atrophy, including VIDD, involves multiple factors such as oxidative stress, inflammation, proteolysis, lipid aggregation, and endoplasmic reticulum stress (Dridi et al., 2020; Picard et al., 2012; Cacciani et al., 2022; Li et al., 2022a; Hyatt et al., 2021; Maes et al., 2014). Muscle atrophy is characterized by reduced muscle strength and the atrophy of muscle fibers. Our study validated that Dex increases markers associated with oxidative stress and proteolysis in the C2C12 cellular model of muscle atrophy. We demonstrated that ENT exerts a protective effect against Dex-induced muscle atrophy by reducing the Dex-induced myotube diameter, improving cell viability, and inhibiting proteolysis and oxidative stress.

In our VIDD model, ENT significantly attenuated the decline in diaphragmatic muscle strength related to mechanical ventilation and mitigated the elevation of markers associated with ventilator-associated muscle atrophy. Additionally, in murine models, we showed that Dex-induced muscle atrophy leads to atrophy of the diaphragm and gastrocnemius muscles, which were ameliorated by ENT treatment. Furthermore, LPS administration led to an increase in the expression of muscle atrophy markers and oxidative stress markers, which was alleviated by ENT. Notably, hyperlipidemic mice exhibited decreased muscle strength, muscle atrophy in the diaphragm, and increased muscle lipid accumulation compared to C57 mice. ENT significantly reduced lipid aggregation in the gastrocnemius muscle, suggesting that its protective effects may be attributed to its ability to suppress lipid accumulation.

However, this study has several limitations. Firstly, the induction of muscle atrophy in C2C12 myotubes by Dex does not entirely replicate the complexity of muscle atrophy observed *in vivo*, which may lead to an under- or overestimation of ENT’s protective effects. Secondly, the inflammation-related muscle atrophy model did not successfully detect changes in inflammatory markers. Additionally, we were unable to investigate the specific signaling pathways involved in the protective effects of ENT on muscle atrophy.

While we have acknowledged these limitations, we also see opportunities for future studies. Investigating the specific signaling pathways involved in ENT’s protective effects is essential. Long-term studies could also be conducted to explore the clinical applications of ENT in preventing or treating muscle atrophy in human populations.

The clinical implications of our findings are significant. By establishing ENT’s potential role in muscle atrophy, we pave the way for future clinical trials. Identifying specific patient populations—such as those experiencing muscle wasting due to aging, chronic illness, or prolonged mechanical ventilation—that could benefit from ENT treatment will be crucial for translational research.

Our findings underscore the potential of ENT as a therapeutic intervention for muscle atrophy across different contexts. By clearly articulating these varying settings of muscle wasting, we hope to establish a foundation for further research into ENT’s mechanisms and its application in clinical settings for muscle atrophy management.

## Conclusion

In summary, our study demonstrate that ENT effectively inhibits oxidative stress and lipid aggregation, thereby exerting a protective effect against muscle atrophy. Additionally, ENT appears to modulate molecular pathways involved in proteolysis and muscle atrophy. Based on these findings, ENT holds promise as a potential pharmacological intervention for treating muscle atrophy.

## Data Availability

The original contributions presented in the study are included in the article/supplementary material, further inquiries can be directed to the corresponding authors.

## Abbreviations

VIDD, ventilator-induced diaphragmatic dysfunction; Dex, dexamethasone; Murf-1, muscle ring finger 1; ENT, entacapone; TC, total cholesterol; HDL-C, high-density lipoprotein cholesterol; TG, triglycerides; LDL-C, low-density lipoprotein cholesterol; GAPDH, Glyceraldehyde-3-phosphate dehydrogenase.
